# Development, optimization and characterization of nanoemulsion loaded with clove oil-naftifine antifungal for the management of tinea

**DOI:** 10.1080/10717544.2021.1879314

**Published:** 2021-02-01

**Authors:** Adel F. Alghaith, Sultan Alshehri, Nabil A. Alhakamy, Khaled M. Hosny

**Affiliations:** a Department of Pharmaceutics, College of Pharmacy, King Saud University, Riyadh, Saudi Arabia; b Department of Pharmaceutical Sciences, College of Pharmacy, Almaarefa University, Riyadh, Saudi Arabia; c Faculty of Pharmacy, Department of Pharmaceutics, King Abdulaziz University, Jeddah, Saudi Arabia; d Faculty of Pharmacy, Department of Pharmaceutics and Industrial Pharmacy, Beni-Suef University, Beni-Suef, Egypt

**Keywords:** Nanoemulsion, tinea, clove oil, naftifine, Box–Behnken, experimental design

## Abstract

Tinea is a common superficial infection caused by keratinophylic fungi called dermatophytes. The objective of the current investigation was to develop and optimize a self-nanoemulsion drug delivery system (SENDDs) using clove oil loaded with naftifine (NF). Clove oil possesses good anti-inflammatory and antifungal properties that can support naftifine action. Box–Behnken designs were used to prepare plain and naftifine loaded SENDDs. The plain SENDDs were evaluated for their globule size. The medicated formulations (NF-CO-SENDDs) were characterized by measuring their globular size, *ex vivo* % NF permeated, level of interleukin-31 in rats, and antifungal activity. The optimum clove oil level was found to be 10–17%, while NF-CO-SENDDs formulations displayed globular sizes ranging from 119 to 310 nm. The statistical design confirmed the synergistic effect of clove oil and NF in the treatment of fungal infections, confirming that the anti-inflammatory effect of clove oil can counteract the side effects of NF. The optimized formulation composed of 14% clove oil, 12.5 mg Naftifine, and prepared with an Smix ratio equaling 3:1, exhibited good antifungal and anti-inflammatory activity, achieving up to 2-, 3-, 5.75-, and 2.74-fold increases in the amount of permeated NF, steady-state flux, permeability, and diffusion coefficients, respectively, compared with a commercial product. Moreover, the optimum formulation revealed an adequate zeta potential value of 28.31 ± 1.37 mV and showed reasonable stability with no or mild signs of skin sensitivity. Therefore, the designed nanoemulsions containing a combination of clove oil and naftifine could be considered promising delivery systems for the treatment of tinea.

## 1. Introduction

Superficial fungal infections, such as tinea, are one of the most prevailing infections worldwide primarily caused by dermatophytes of the genera *Epidermophyton*, *Microsporum*, and *Trichophyton* (Branscomb, 2005). Such aerobic pathogenic fungi can grow and colonize on keratinized tissues like skin, hair and nails, owing to their ability to produce numerous proteolytic enzymes that can digest keratin (Branscomb, 2005). These infected keratinized tissues provide the desired temperature, pH, and nitrogen conditions to meet the nutritional needs of dermatophytes; therefore, such infections are restricted to superficial cutaneous tissues and rarely extend to deeper subcutaneous ones (Bottone, 2006). Although several antifungals have been reported to have essential activity against dermatophytes, such infections often relapsed after the medication was stopped since these organisms established a tolerance to prolonged treatment (Mukherjee et al., 2003).

Dermatophytosis is usually treated through oral or topical pathways or a combination of both, considering the site, infection extent, severity, and causative organism (Rotta et al., 2012). Principally, the topical route is thought to be the first-line treatment for superficial and uncomplicated infections due to its high efficacy, good ability to localize drugs at the infection site, and low chance for systemic absorption, hence offering much fewer side effects (van Zuuren et al., 2015).

Naftifine, a primary topical antimycotic drug with allylamine structure, is active against a broad spectrum of dermatophytes belonging to *Trichophyton* and *Microsporum* spp. and has shown good activity against *Candida* and *Aspergillus* spp. (Cuenca-Estrella et al., 2006). It is believed to exert a fungicidal effect through squalene epoxidase inhibition in fungi, thus diminishing ergosterol biosynthesis (Monk & Brogden, 1991). In contrast to other antifungal drugs like azoles, naftifine is highly selective to ergosterol biosynthesis and does not affect drug metabolism in the liver even if a significant portion reaches the systemic circulation (Lee et al., 2007). Although naftifine is well tolerated, it was reported to cause some mild inflammations and stinging sensation, which might affect patient compliance (Altmeyer et al., 1990).

Naftifine is a topical, synthetic allylamine derivate similar to terbinafine, its molecular weight is 287.4, its logP equals 5, it has very poor aqueous solubility (0.000229 mg/mL) and pKa equal 9.08 (Chen et al., 2002)

Eugenol is a phenolic compound that accounts for 45–90% of clove essential oil (Zhang et al., 2009) and has exhibited several pleiotropic activities, such as anti-inflammatory (Kim et al., 2003), anticancer, bactericidal (Hamed et al., 2012), antifungal (Darvishi et al., 2013), anesthetic (Tsuchiya, 2017), and analgesic effects (Baldisserotto et al., 2018). Its anti-inflammatory effect might be ascribed to its ability to block the nuclear factor-kappa B (NF-κB) signaling pathway, which is responsible for producing the inflammatory cytokines, namely interleukin-6 (IL-6), interleukin-1β (IL-1β), and tumor necrosis factor-α (TNF-α) (Zhang et al., 2013). More importantly, eugenol offers good activity against dermatophytes due to its interaction with the fungal envelope, leading to the leakage of cells’ essential elements and eventually cell death (Lee et al., 2007). Furthermore, several investigations have suggested that eugenol also offers a transdermal penetration-enhancing effect (Mutalik & Udupa, 2003). Based on the above-mentioned findings, clove oil appears to be a promising support therapy in treating topical skin infections like tinea.

An exemplary drug delivery system should offer maximum therapeutic effect with minimum toxicity (Montenegro et al., 2017). Nano-sized drug delivery systems have been considered a good substitute for conventional ones (Abou-Taleb et al., 2018; Alkhalidi et al., 2018), such as nanoemulsions (NE) systems that consist of 20–500 nm-sized nano-droplets stabilized by surfactants. Frequently, NE can be formulated as water-in-oil (W/O), oil-in-water (O/W) and also multiple emulsions (W/O/W) (Esmaeili et al., 2016; Hosny et al., 2019) systems, which exhibit several strength points compared to conventional emulsions. These advantages include: (1) the ability to effectively transport drugs to target sites owing to their very large surface area offered by the very small size of their droplets; (2) the capacity to guard against hydrolysis and enzymatic degradation of drugs; (3) enhanced drug loading, drug solubility and bioavailability; (4) decreased intra-patient variations, and (5) ability to attain controlled drug release (Sigward et al., 2013). Due to such advantages, NE provides an ideal platform for drug delivery systems.

Nanoemulsions are usually stabilized by employing various surfactants and co-surfactants that form a protective fil around oil globules, preventing their coalescence and NE separation (Jaiswal et al., 2015). Quaternary ammonium compounds like cetylpyridinium chloride (CPC) were reportedly used as surfactants in nanoemulsions due to their stabilizing effect (Barney et al., 2006) and antimicrobial action against a broad spectrum of bacteria and fungi (Rawlison et al., 2008). Moreover, it is considered more effective against aerobic and anaerobic microorganisms with only minimal concentrations compared to other antiseptics like chlorhexidine (Uerra et al., 2019).

The production of an effective drug delivery system requires a rational mixture of drugs and excipients; therefore, optimizing the formula composition is essential to achieve optimal quality. To obtain this goal, Design of Experiments (DoE) methodologies have been recently applied, in which the “best solution” can be achieved through fewer experiments to attain an optimal formulation (Singh et al., 2011). Moreover, DoE offers a better understanding of the formula composition and tracking of problems that might emerge during experimentation. Furthermore, DoE can help in assigning the more important input variables through certain screening techniques (Dhawan et al., 2011) and can uniquely anticipate a formulation’s performance prior to its preparation, hence saving effort, time, supplies, and cost (Huang et al., 2011). Considering these objectives, the current study was conducted to develop a clove oil-based nanoemulsion loaded with naftifine HCl as a topical treatment for superficial tinea infections.

## 2. Materials and methods

### 2.1. Materials

Naftifine was acquired as a generous gift from Pharco Pharmaceutical Company, Egypt. (Alexandria, Egypt). Cetylpyridinium chloride was acquired as a generous gift from Saudi Drugs and Medical Instruments Company (SPIMACO), in (Qassim, Saudi Arabia). Clove oil was purchased from Avanti Polar Lipids (Alabaster, AL, USA). Diethylene glycol monoethyl ether (Transcutol^®^) was kindly provided by Gattefossé (Lyon, France) High-performance liquid chromatography grade methanol and acetonitrile were obtained from Merck (Darmstadt, Germany). Chloroform, absolute ethanol, and phosphate buffer pH 7.4 were purchased from Fisher Scientific UK (Loughborough, Leicestershire, UK). All other reagents and chemicals were of analytical grade.

### 2.2. Methods

#### 2.2.1. Experimental design and optimization of self-nanoemulsion formulations

Two response surface Box–Behnken designs were adopted during the current investigation using Design-Expert^®^ software v. 12.0.6.0 (Stat-Ease, Inc., Minneapolis, MN, USA). The first design was developed to investigate the effects of independent variables (i.e. (A) clove oil %, (B) the Smix ratio of surfactant to co-surfactant, and (C) amount of water added on the globule size of plain self-nanoemulsion formulations (SNEDDS). The second design was created to explore the effect of the independent variables, including (A) clove oil %, (B) the amount of NF in milligrams, and (C) the Smix ratio of surfactant to co-surfactant in the prepared medicated nanoemulsions. The investigated dependent responses were the globule size of the prepared SNEDDS (Y1), *ex vivo* % of naftifine permeated through rat skin (Y2), the zone of inhibition against *Trichophyton rubrum* (Y3), and interleukin-31 level (Y4). The independent factors and determined responses are shown in Tables 1–3.

**Table 1.** Independent variables and their levels, along with dependent variables and their constraints in the Box–Behnken designs of plain and medicated nanoemulsion formulations.

	Levels	
	Low	High
Plain SNEDDS		
Independent variables		
A = Clove oil %	10%	20%
B = Smix ratio (surfactant: co-surfactant) %	40%	60%
C = Water %	20%	50%
Dependent variables	Constrains	
Y1 = Droplet size (nm)	Minimize	

	Levels		
Independent variables	−1	0	1
A = Clove oil %	10%	13.5%	17%
B = Naftifin amount (mg)	10	20	30
C = Smix ratio (surfactant: co-surfactant)	1:1	1:2	1:3
Medicated SNEDDs			
Dependent variables	Constrains		
Y1 = Droplet size (nm)	Minimize		
Y2 *Ex vivo* % naftifine permeated	Maximize		
Y3 = zone of inhibition against *Trichophyton rubrum* (mm)	Maximize		
Y4 = Interleukin-31 Level (pg/mL)	Minimize		

**Table 2.** Box–Behnken design responses of plain NE formulation.

Run	A	B	C	Response 1	
	Clove Oil %	Smix %	Water%	Globule Size (nm)	PDI
1	0.15	50.0	35.0	142	0.23
2	15.0	60.0	24.9	147	0.19
3	14.6	48.4	36.8	138	0.10
4	20.0	50.3	29.6	195	0.33
5	10.0	57.0	32.9	85	0.35
6	20.0	44.3	35.6	185	0.25
7	10.0	47.7	42.2	96	0.11
8	13.7	42.0	44.2	123	0.35
9	20.0	60.0	20.0	175	0.14
10	10.0	57.0	32.9	86	0.29
11	19.2	40.0	40.7	167	0.30
12	15.0	60.0	24.9	146	0.17
13	19.2	40.0	40.7	166	0.11
14	14.6	48.4	36.8	136	0.24
15	15.3	54.9	29.7	153	0.34
16	14.6	48.4	36.8	136	0.16
17	20.0	55.1	24.8	200	0.26
18	10.0	40.0	50.0	145	0.30

**Table 3.** Box–Behnken design responses of NF-CO-SENDDs.

Run	A	B	C	Y1	Y2	Y3	Y4	PDI
	A:Clove Oil %	B:Naftifine amount (mg)	C:Smix ratio	Globule size (nm)	*Ex vivo* % naftifine permeated (%)	Zone of inhibition against *Trichophyton rubrum* (mm)	Interleukin-31 Level (pg/mL)	
1	1	−1	−1	164	56	5	120	0.12
2	−1	1	−1	200	34	12	750	0.23
3	−1	−1	−1	119	52	3	190	0.22
4	1	1	−1	295	52	13	340	0.30
5	0	0	0	171	65	16	300	0.15
6	0	0	−1.68	161	69	10	255	0.14
7	−1.68	0	0	128	18	12	590	0.32
8	−1	−1	1	141	44	14	200	0.40
9	1	−1	1	186	52	15	175	0.19
10	0	−1.68	0	146	60	6	100	0.25
11	0	0	0	172	63	16	300	0.36
12	0	1.68	0	310	41	22	680	0.31
13	0	0	1.68	178	59	21	385	0.21
14	1	1	1	289	44	24	385	0.22
15	−1	1	1	190	25	22	800	0.17
16	1.68	0	0	255	35	19	260	0.10
17	0	0	0	173	62	17	300	0.20

#### 2.2.2. Self-nanoemulsion preparation

The development of NF-loaded nanoemulsions was performed in two steps. The first step included the formation of the plain SNEDDS, in which 10–20% clove oil concentrations were mixed with 40–60% surfactant and cosurfactant mixture (Smix) and 20–50% water. The surfactant and co-surfactant were mixed in three different ratios (1:1, 2:1, and 3:1), according to the first design. In the second step, clove oil concentrations of 10, 13.5, or 17% were mixed with 90, 86.5, or 83% Smix, respectively. The active ingredient, NF, was mixed with the plain SNEDDS with the aid of sonication in concentrations of 10, 20, or 30 mg/g according to the second design, as is shown in Table 1.

#### 2.2.3. Globule size measurements

The droplet size of either plain CO-SNEDDS or NF-CO SNEDDS was measured by diluting 200 μL SNEDDS with 800 μL purified water in a volumetric flask. The diluted samples were vigorously agitated, and then 200 μL was withdrawn and used to measure the droplet size on a Microtrac^®^ zeta track particle size analyzer (Microtrac, Inc., Montgomeryville, PA, USA) (Hosny et al., 2020).

#### 2.2.4. *Ex vivo* permeation study

The *ex vivo* permeation study was performed following a previously published method (Salem et al., 2020). The NF permeation across rat skin from nanoemulsions was assessed using Franz cells with a diffusion area of 5 cm^2^, according to a previously reported method. 8-weeks-old Wistar albino rats, weighing 150–200 g, were used in this study. Following animal sacrifice, shaved abdominal skin was excised, separated from underlying connective tissues using a scalpel, and finally used as a model permeation membrane. The obtained membranes were mounted between the donor and receptor compartments of Franz diffusion cells so that the stratum corneum was facing the donor, while the dermal skin side was facing the receptor compartment. Nanoemulsions (equivalent to 20 mg NF) were placed in the donor compartment of the Franz cells, and the receptor compartment was filled with 12 mL phosphate buffer saline (PBS, pH 7.4). The medium was stirred at 100 rpm, and the temperature was kept at 37 ± 0.5 °C during the experiment. 1-mL samples were withdrawn at fixed time intervals of 0.5, 1, 2, 4, 8, 12, and 24 h, then the receptor media was replenished with equal volumes of fresh media to maintain sink conditions.

Collected samples were then analyzed for their NF content using high-performance liquid chromatography (HPLC) in which a mixture of acetonitrile, tetrahydrofuran, and tetramethyl-ammonium hydroxide with a ratio of 62:10:28, respectively (pH 7.8), was used as the mobile phase that was pumped at a fixed flow rate of 1.2 mL/min. The measurements were performed at a maximum wavelength of 240 nm using an Agilent 1260 Infinity Diode array detector VL (G131SD) and injection volume of 100 μL.

Limit of detection of and linear range naftifine were 0.0201 μg/mL and 0.080 μg/mL respectively.

The peak areas of Naftifine HCl were plotted against Naftifine HCl concentrations. The least square line regression analysis was used to determine the slope, Y-intercept, and the correlation coefficient of the standard plots.

#### 2.2.5. Antifungal activity evaluation

The antifungal activity of NF-CO-SENDDs against *Trichophyton rubrum* was assessed using a slightly modified disk diffusion susceptibility method (Mahtab et al., 2016). According to the method recommended by the Clinical and Laboratory Standards Institute, a suspension of *Trichophyton rubrum* was made to 0.5 McFarland turbidity standard (106 CFU/mL) by employing a hemocytometer. An inoculum of 1 mL, from a suspension of 104 CFU/mL, was spread on the surface of Mueller–Hinton agar plates using sterile loops. Disks of 10-mm diameters containing NF-CO-SENDDs were then placed onto the inoculated agar plates using sterile forceps. The prepared plates were incubated at 37 °C for 24 h, and growth inhibition zone diameters (where fungal growth markedly decreased) were measured for each NF-CO-SENDDs formulation.

#### 2.2.6. Interleukin-31 measurements

Male Wistar albino rats averagely weighing 250 ± 10 gm were used in the study. 150 mg of each formulation containing 3.5 mg of NF were applied topically to the saved animals’ skin. Animals received treatment once daily for 14 days. At the end of the experiment, blood samples of 1 mL were withdrawn from animals’ tail vein and placed in edita tubes, then the samples were centrifuged at 4000 rpm for 10 min, supernatant (i.e. serum) was removed and kept at −20 °C for further investigations.

Concentrations of IL‐31 serum levels were determined using an enzyme‐linked immunosorbent assay (ELISA) technique. Rat IL‐31 Quantikine M‐assay (R&D, Minneapolis, MN, USA, Catalog no. M1300C) was carried out according to the manufacturer’s instructions. A monoclonal antibody specific for rat’s IL-31 was pre-coated onto a microplate, and goat‐anti‐rat IgG (Jackson Immuno, Catalog no. 115‐035‐166) was employed as the detection antibody. A microtiter plate reader was used for optical density determination at 450 nm, and concentrations of IL-31serum levels were expressed in pg/mL (Grimstad et al., 2009).

#### 2.2.7. Optimization and evaluation of the selected formulation

##### 2.2.7.1. Experimental model evaluation

Degrees of freedom, *F*-ratio, and *p*-value for all factors and their interactions were determined to analyze the variance of calculated models for the measured responses and select the model that best fit the obtained data. Specifically, *p*-values less than .05 indicate the significance of the model. Moreover, the determination coefficients, predicted (*R*^2^), adjusted (*R*^2^), and CV% values were used to determine the model fitness. In the current investigation, the criteria used for identifying the optimized medicated NF-CO SENDDs included minimum globule size and IL-31 levels and maximum *ex vivo* % naftifine and zone of inhibition against *Trichophyton rubrum*.

##### 2.2.7.2. Characterization of the optimized NF-loaded nanoemulsion formulation

The optimum formulation was fabricated and characterized by determining its globule size, IL-31 levels, *ex vivo* % naftifine permeated across skin, and zone of inhibition against *Trichophyton rubrum* and compared against that of commercial cream. For further evaluation, NE was subjected to skin sensitivity and stability studies. The *ex vivo* diffusion study for optimum formulation was performed as previously mentioned, with additional calculation of the diffusion coefficient (D), permeability coefficient (P), steady-state transdermal flux (Jss), and enhancement ratio (ER).

###### 2.2.7.2.1. Zeta potential (ZP) measurements

An electrophoretic mobility study was employed to assign the surface charges of the optimum formulation using a Malvern Zeta sizer (Malvern instruments, Malvern, UK) at a temperature of 25 °C and a fixed angle of 90° (Abdelbary et al., 2017).

###### 2.2.7.2.2. Skin sensitivity test

The test rats were held in laboratory cages and allowed free access to water and food. Animals were acclimatized for a period of 14 days prior to experimentation under standard conditions of 55 ± 5% relative humidity, 25 ± 1 °C temperature, and a 12-h dark and 12-h light cycle. Rats were handled and tended to according to the Animal Ethics Committee guidelines, Beni-Suef Clinical Laboratory Center, Beni-Suef, Egypt. Researchers followed the guidelines set forth in the Declaration of Helsinki and its “Guiding Principles in the Care and Use of Animals” (NIH Publication No. 85-23, 1985 revision), complying to ethical approval of the protocol before starting the experiment (Approval No. M12-11-2020).

The dorsal skin of rats (4 × 4 cm^2^) was carefully shaved with an electric clipper so as not to cause any damage. Three animal groups, each composed of 6 rats, were employed in the study. The first group was treated with normal saline and served as the control, the second group received the optimized formulation, and the third group received the commercial product. The tested formulations were applied twice a day for a period of 3 days. Quantification of 5 inflammation signs which were itching, erythema, papule, flakiness, and dryness were used in this study to determine the degree of skin sensitivity. Each parameter was assigned a score from 0 to 3, where 0 represents no sign of inflammation, while 4, represents the severe signs of inflammation (Mahtab et al., 2016).

###### 2.2.7.2.3. Stability studies

The physical stability of the optimum nanoemulsion formulation was tested following a previously reported method with a slight modification (Shafiq-un-Nabi et al., 2007). The optimized formulation was subjected to three freeze-thaw cycles between −20 °C and +25 °C, stored at each temperature for 48 h, and finally examined for their emulsification ability, precipitation, pH, and globule size.

## 3. Results and discussion

Dermatological and topical dosage forms are usually designed to easily deliver active agents across certain skin areas. Concerning the topical route, poor drug permeability contributes to prolonged treatment, hence leading to low patient adherence and high cost of treatment. Nano-sized drug delivery systems are usually used to overcome such limitations. These delivery systems permit high drug concentrations to penetrate the skin through an intercellular pathway (Singh & Ahuja, 2004) and depot drugs in the epidermis and stratum corneum. Nanoemulsions are one of the most extensively-used topically applied drug delivery systems to treat skin conditions, such as fungal infections.

### 3.1. Box–Behnken design analysis

**Figure 1.**
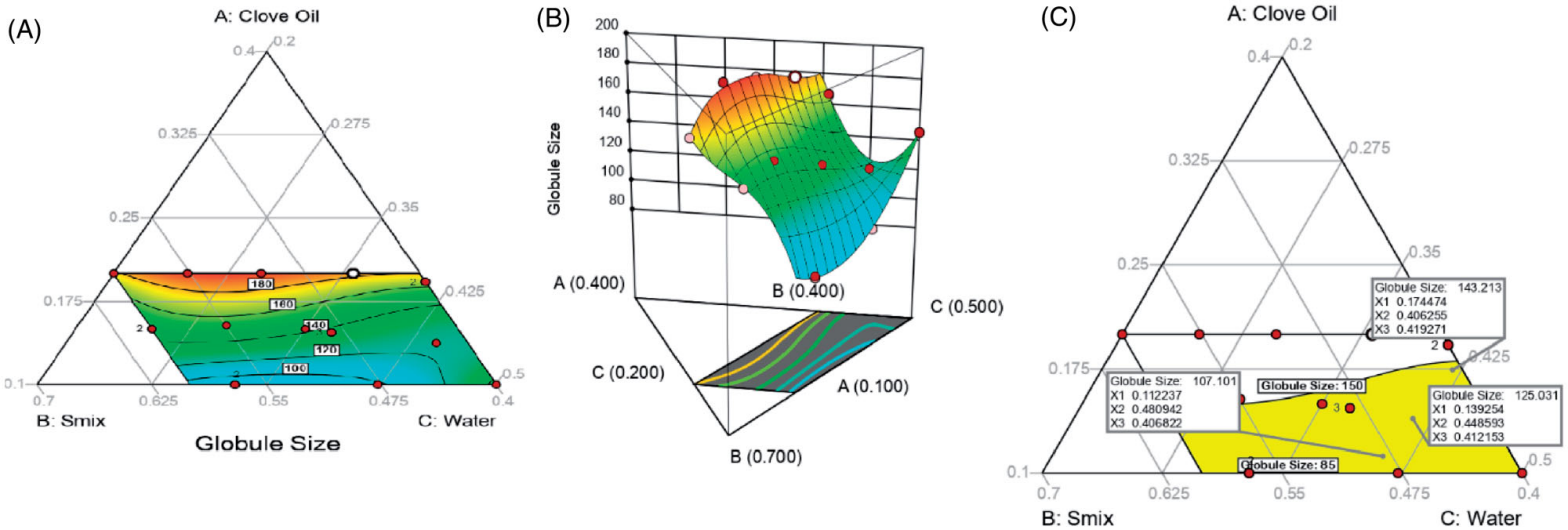
(A) Contour plot, (B) 3D surface plot, and (C) overlay plot showing the effects of different independent variables on the globule size of plain nanoemulsion.

**Figure 2.**
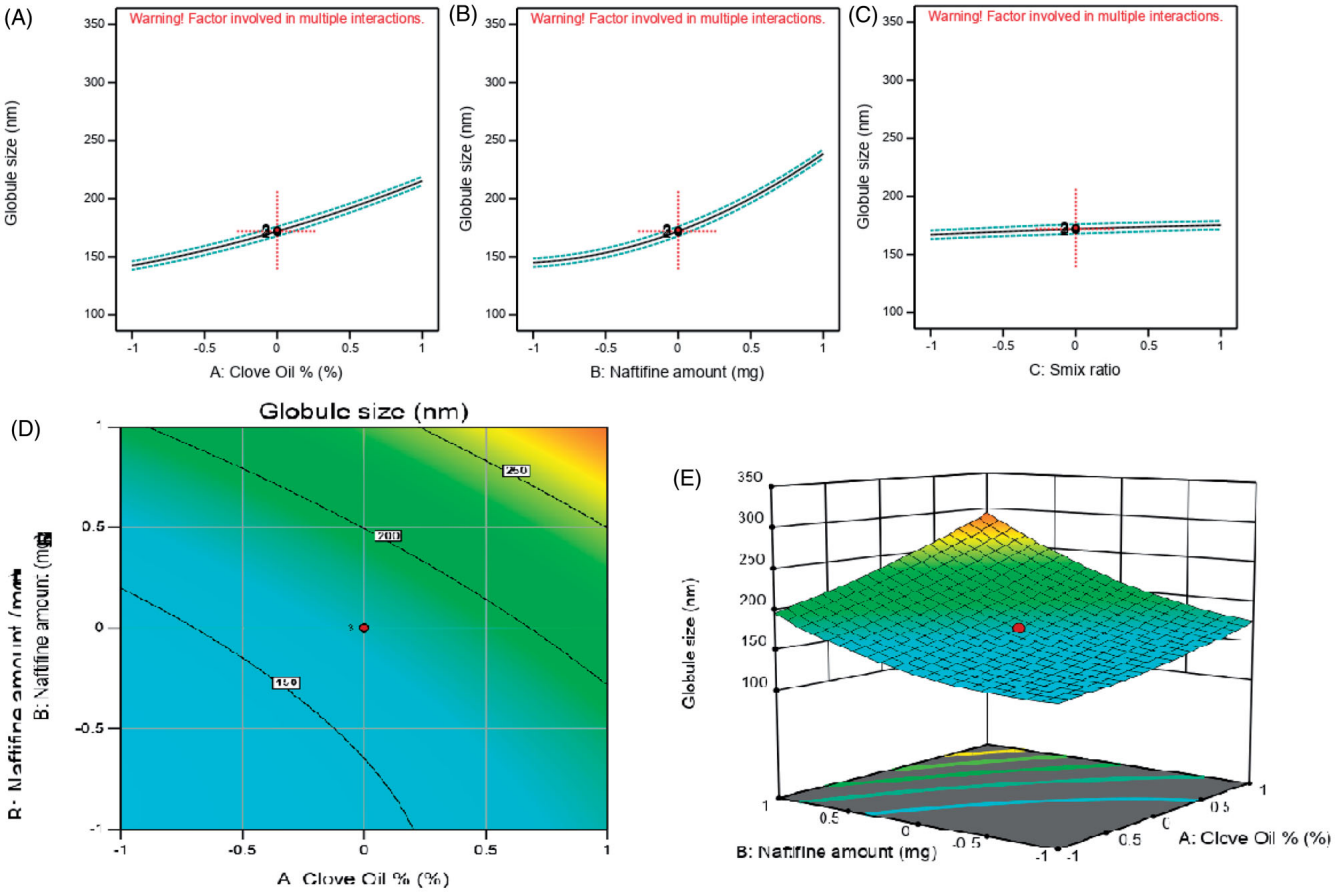
Main effect diagram, contour, and 3D response surface plots representing the effect of the studied variables on droplet size (Y1). (A) Main effect diagram of clove oil (%); (B) main effect diagram of naftifine amount (mg); (C) main effect diagram of Smix ratio (%); (D) contour plot showing the effect of clove oil and naftifine on globule size; (E) 3D surface plot representing the effects of the clove oil % and naftifine amount (mg) on droplet size.

**Figure 3.**
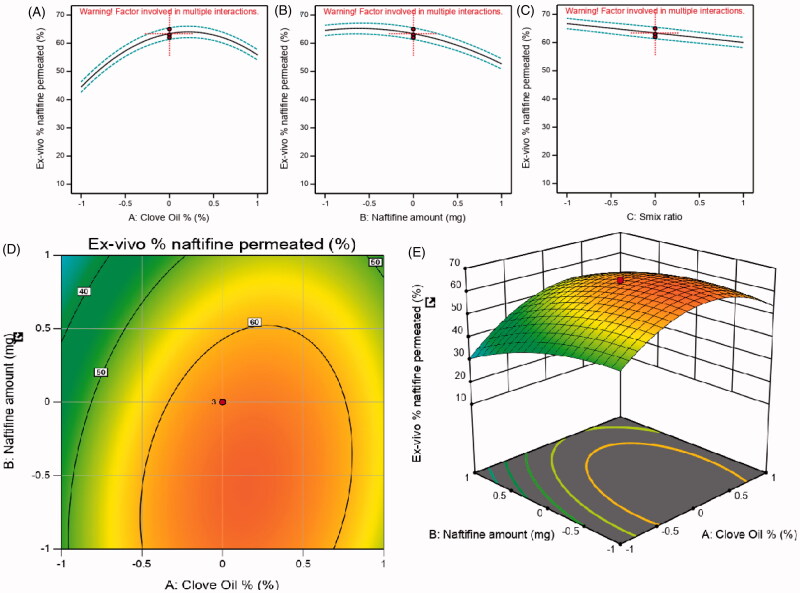
Main effect diagram, contour, and 3D response surface plots representing the effect of the studied variables on *ex vivo* % naftifine permeated (Y2). (A) Main effect diagram of clove oil (%); (B) main effect diagram of naftifine amount (mg); (C) main effect diagram of Smix ratio (%); (D) Contour Plot showing the effect of clove oil and naftifine on *ex vivo* % naftifine permeated; (E) 3D Surface plot representing the e effects of the clove oil % and naftifine amount (mg) on *ex vivo* % naftifine permeated.

**Figure 4.**
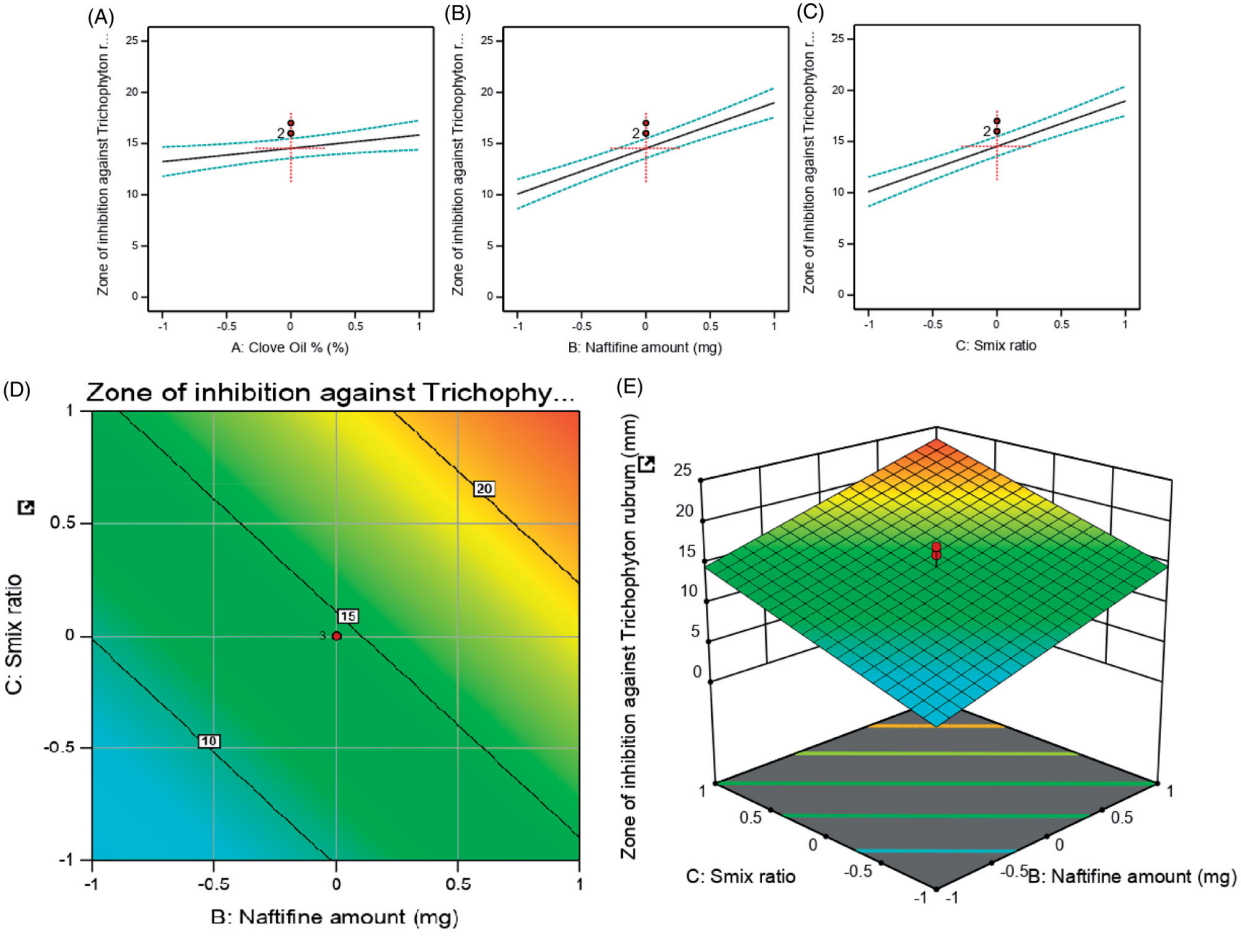
Main effect diagram, contour, and 3D response surface plots representing the effect of the studied variables on the zone of inhibition against *Trichophyton rubrum* (Y3). (A) Main effect diagram of clove oil (%); (B) main effect diagram of naftifine amount (mg); (C) main effect diagram of Smix ratio (%); (D) contour plot showing the effect of clove oil and naftifine on zone of inhibition against *Trichophyton rubrum*; (E) 3D surface plot representing the effects of the clove oil % and naftifine amount (mg) on zone of inhibition against *Trichophyton rubrum*.

**Figure 5.**
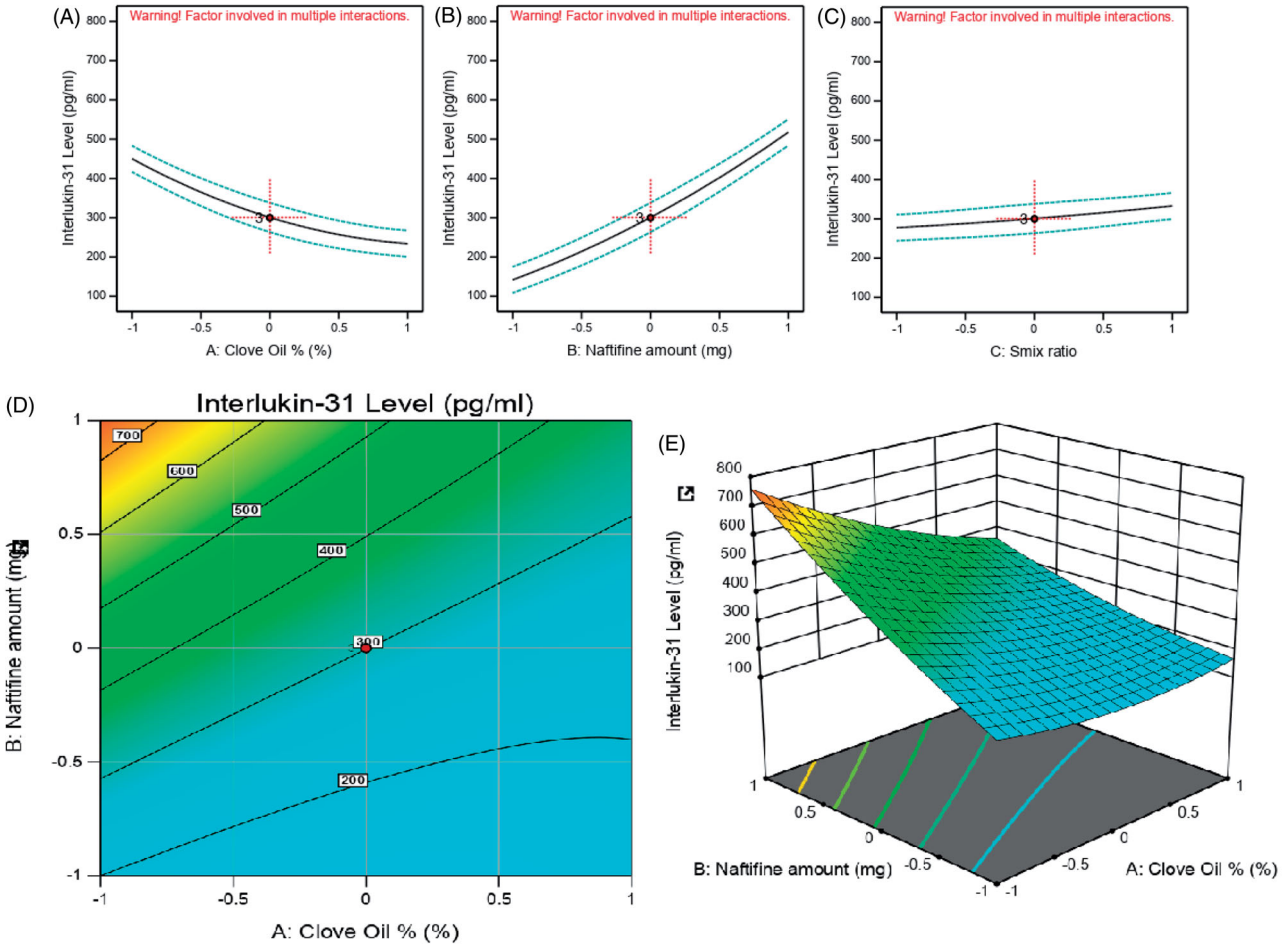
Main effect diagram, contour, and 3D response surface plots representing the effect of the studied variables on IL-31 level (Y4). (A) Main effect diagram of clove oil (%); (B) main effect diagram of naftifine amount (mg); (C) main effect diagram of Smix ratio (%); (D) contour plot showing the effect of clove oil and naftifine on IL-31 levels; (E) 3D surface plot representing the effects of the clove oil % and naftifine amount (mg) on IL-31 levels.

The two experimental designs were created using Design-Expert software (12.0.6.0, Stat-Ease, Inc., Minneapolis, MN, USA). The first design was used to determine the effect of each independent variable and its interactions on the globular size of plain NE and allocate the optimum oil amount to be used in drug-loaded NE. The second design was performed to study the effect of each independent variable and its influence on the globular size of medicated NE, *ex vivo* % of naftifine permeated, zone of inhibition against *Trichophyton rubrum*, and interleukin-31 level. Analysis of variance (ANOVA) and *F*-values at a 95% confidence interval (*p* < .05) were employed to statistically test the validation of the selected model. Checkpoint analysis was used to check the accuracy and validity of the obtained mathematical models in terms of the predictions of dependent responses. The main effect diagrams, 3D-surface response, contour, and overlay plots of the desired responses relative to the optimal region in which the optimal nanoemulsions can be obtained were developed (Figures 1–5). Finally, desirability values were determined, and the predicted and actual parameters were compared to evaluate the formulation.

### 3.2. Formulation and characterization of plain nanoemulsion

#### 3.2.1. Nanoemulsion droplet size and polydispersity index (PDI)

Concerning topical delivery, NE physicochemical properties predominantly indicate the effectiveness of a developed formulation (Singh et al., 2005). Globule size is the most important NE character as it could differentiate the developed emulsions into micro-emulsions or nanoemulsions, whereby the formation of NE with the lowest globule size is highly desired. As shown in Table 2, the droplet size of the currently developed plain NE was in the range of 85–195 nm with an acceptable PDI between 0.1 and 0.35, which indicates fair homogeneity, adequate size distribution, and acceptable NE stability.

The quadratic model of polynomial analysis was obtained to analyze the droplet size values and further explore the significant effect of (A) clove oil %, (B) Smix ratio, and (C) water % on the NE globule size. The suggested quadratic model acquired an adjusted *R*^2^ of 0.9984, which was very close to the predicted *R*^2^ of 0.9829. ANOVA analysis of the obtained data yielded the following equation:

Globule size= +1141.45 A +152.47 B +144.91 C −1379.88 AB−1338.49 AC −261.02 BC −2787.09A²BC+4130.83AB²C+2540.86 ABC²

As could be seen from the equation, all investigated variables had a significantly positive effect on NE droplet size with a *p*-value <.0001. However, the amount of oil was determined as the most pertinent parameter as it exhibited the largest coefficient (1141.5) compared to the Smix ratio (152.47) and water amount (144.91). In addition, the interaction between parameters involving (A) clove oil %, such as that with (AB) Smix ratio and (AC) water %, exhibited larger coefficient values (1379.88 and 1338.49, respectively) compared to the interaction of (BC), which exhibited a coefficient value of 261.02. The increase in globule size corresponding to increased clove oil amount could be explained by considering that an increase in oil amount would decrease the Smix ratio. Subsequently, this would decrease the capacity of the surfactant and co-surfactant to downsize the oil droplets; thus, larger oil globules would be obtained, which is similar to previously reported results (Cevc & Vierl, 2010; Okur Apaydin et al., 2011). Moreover, increasing the water amount would increase the volume of the aqueous phase and yield larger droplets.

Figure 1 displays the contour, 3D-surface, and overlay plots that demonstrate the effect of the independent variables on plain NE droplet size. Contour and 3D-surface plots indicate that the globule size of plain NE mainly depends on the oil level in nanoemulsions, and the overlay plot reveals that the globule size was around 100 nm with a 10% oil concentration and was elevated to 143 nm when oil increased to 17%. Based on these results, the optimum range of clove oil that would yield a formulation with acceptable size was found between 10 and 17%; consequently, these oil levels were used to further prepare medicated NE in the second step of this research.

### 3.3. Formulation and characterization of medicated nanoemulsions

#### 3.3.1. Nanoemulsion droplet size and polydispersity index (PDI)

As shown in Table 3, globule size of the medicated NE ranged 119–310 nm with PDI values between 0.1 and 0.4, providing supportive evidence of good formulation stability, homogeneity, and size distribution.

The obtained globule size data were then subjected to a quadratic model of polynomial analysis. The experimental design showed the investigated model’s efficiency to determine the significant effect of (A) clove oil amount, (B) NF amount, and (C) Smix ratio on medicated NE globule size. The chosen model achieved an adjusted *R*^2^ of 0.9973 and predicted *R*^2^ of 0.9913, as shown in Table 4. Data analysis by ANOVA resulted in the following equation:

Globule size= +171.99+36.44 A +46.85B +4.14C +13.00AB+0.5000AC−7.50BC +6.94 A²+19.84B²−0.8416C²

**Table 4.** Regression analysis results for Y1, Y2, Y3, and Y4 responses.

Dependent variables	*R* ^2^	Adjusted *R*^2^	Predicted *R*^2^	*p*-Value	*F*-value	Adequate precision
Y1	0.9988	0.9973	0.9913	.0001	184.78	480.27
Y2	0.9953	0.9893	0.9714	.0001	326.04	97.03
Y3	0.9283	0.9118	0.8777	.0001	56.13	74.16
Y4	0.9930	0.9840	0.9460	.0001	110.8	40380.18

From the above equation, it could be deduced that the (A) amount of oil and (B) amount of drug had a higher positive effect on NE globule size than (C) the Smix ratio, as (A) and (B) exhibited higher coefficient values (36.44 and 46.85, respectively) compared to (C) with a coefficient value of 4.14. The superior effect of oil and drug amounts on droplet size was further confirmed by the main effect diagram in Figure 2, which revealed the high impact of changing the oil and drug amounts on droplet size. Furthermore, the previous equation showed that the interaction of clove oil and NF amount (AB) had the most significant effect on NE globule size compared to other interactions (i.e. AC and BC). Increasing the clove oil amount could have allowed a greater incorporation of the drug and, hence, result in droplets with larger diameter. Moreover, there was a corresponding decrease in the Smix ratio when the oil amount was increased, which led to the decreased ability of Smix to downsize the oil droplets and increased globule size. Comparatively, a higher NF amount might have caused swelling of the droplets and, thus, larger emulsion droplets. Similar results have been reported in the literature (Sakeena et al., 2011; Okur et al., 2020).

Figure 2 illustrate the main effect diagrams, 3D surface response, and contour plots, which reveal the effect of the studied factors on medicated NE droplet size.

#### 3.3.2. *Ex vivo* permeation study of naftifine loaded NE

*Ex vivo* permeation studies usually effectively indicate the performance of a drug and its ability to overcome natural skin barriers like the stratum corneum (SC). The *ex vivo* % of naftifine permeated from drug loaded-NE formulations across rat skin fluctuated between 18–69%, as seen in Table 3. The collected data were used to prepare a quadratic model for polynomial analysis. The Box–Behnken design analysis demonstrates the effectiveness of the model to explore the effect of (A) clove oil amount, (B) NF amount, and (C) Smix ratio (C) on the % naftifine that permeated across the skin of rats.

The suggested statistical model exhibited an adjusted and predicted *R*^2^ of 0.9893 and 0.9714, respectively, which were obviously in close agreement, as shown in Table 4. The following equation was obtained after data analysis using ANOVA:

Ex vivo % naftifine permeated= +63.41+5.68A−5.93B −3.35C +3.12AB+0.6250AC−0.6250BC−13.27A²−4.79B

As indicated in the above equation, (A) clove oil amount had a significantly positive effect on % naftifine permeated, while (B) NF amount and (C) Smix ratio acquired a significantly negative effect on the same parameter. The increase in % NF permeated observed with increased clove oil amount might be attributed to the penetration enhancement characteristics of eugenol, the primary component of clove oil (Chaieb et al., 2007). It was previously reported that eugenol, like many other essential oil components, could interact and disrupt the barrier of SC without damaging the underlying tissues and, hence, promote drug penetration across the skin (Ahad et al., 2016). The inverse relationship between Y_2_ and NF amount and Smix ratio could be ascribed to their effect on NE globule size, since it was observed that the increase in these two factors will result in larger droplets. Consequently, a smaller surface area will be available for drug permeation, leading to decreased % NF permeated across skin, which is similar to findings reported in literature (Hosny et al., 2020).

Although clove oil amount displayed a positive effect on droplets size, its skin penetration enhancement ability overcome limitation of NF permeability. Figure 3 presents the main effect diagrams, 3D surface response, and contour plots, demonstrating the effect of the studied factors on % NF permeated across the skin.

#### 3.3.3. Assessment of antifungal activity of NF-loaded nanoemulsions

The antifungal activity of drug-loaded nanoemulsions was assessed against the dermatophytic fungus *Trichophyton rubrum* by measuring the inhibition zone of fungal growth in plates treated with NE formulations. As shown in Table 3, the diameter of the inhibition zone of *Trichophyton rubrum* equivocated between 5 and 24 mm. Then, based on the inhibition zones against *Trichophyton rubrum*, a linear model of polynomial equations was prepared to test the effect of the independent variables on the measured diameters of inhibition zones.

The model’s predicted *R*^2^ of 0.8777 was in a reasonable agreement with the adjusted *R*^2^ of 0.9118, as presented in Table 4. Data analysis using ANOVA yielded the following equation:

Zone of inhibition against Trichophyton rubrum=+14.53+1.30A+4.46B+4.43C

This equation implies that all the used independent variables exerted a significantly positive effect on the inhibition zone against *Trichophyton rubrum*. In other words, increasing the amount of any of the three tested factors will increase the inhibition zone diameter; however, (B) NF amount and (C) Smix ratio showed a significant effect on the response (*p*-value < .0001) compared to (A) clove oil % with a *p*-value <.02.

The antifungal activity of clove oil against the tested fungus could be attributed to its eugenol content, which agrees with numerous other studies. As mentioned in literature, eugenol could induce leakage of potassium from fungal cells, thus inhibiting the uptake and use of energy by these cells, leading to envelop disruption, and finally inducing cell death (Chee & Lee, 2007).

Moreover, the fungicidal effect of NF is thought to be conducted through the inhibition of fungal squalene epoxidase, inhibiting ergosterol biosynthesis (Monk & Brogden, 1991). The significant anti-fungal activity of NF against the tested fungus could be attributed to its high selectivity to ergosterol biosynthesis, unlike other anti-fungal drugs, such as azoles (Lee et al., 2007).

The observed significant anti-fungal activity of the Smix could be ascribed to its cetylpyridinium chloride (CPC) content. Quaternary ammonium salts like CPC possess antimicrobial effects through various mechanisms (Sreenivasan et al., 2013), for instance, by disrupting cell membrane lipid bilayers and ultimately causing cellular content leakage., and finally cause cellular content leakage (Garcia-godoy, 2014). Moreover, exposure to CPC for longer periods could lead to additional destruction of intracellular materials, indicating autolysis (Evandro et al., 2008). Figure 4 displays the main effect diagrams, 3D surface response, and contour plots, which revealed the effect of the studied factors on the zone of inhibition against *Trichophyton rubrum*.

#### 3.3.4. Interleukin-31 level measurements

IL-31, a T-cell derived cytokine, may be involved in provoking some epithelial responses in atopic skin inflammation, such as redness, pain, and itching, that characterize some allergic reactions (Dillon et al., 2004).

The obtained data of IL-31 serum levels were used to prepare a quadratic model for polynomial analysis following the Box–Behnken design in order to determine the impact of the studied factors on IL-31 serum levels. The model revealed a predicted *R*^2^ of 0.9460, which was in reasonable accordance with the adjusted *R*^2^ of 0.9840, as seen in Table 4. Data analysis by ANOVA produced the equation below:

Interlukin−31 Level=+300.84−108.00A+187.85B+27.72C−91.25AB+5.00AC+3.75BC+41.29A²+28.91B²+4.17C²

The measured IL-31 serum levels fluctuated between 100 and 800 pg/mL in the test rats, as presented in Table 3. From the above equation, it was noticed that (A) clove oil % presented a significantly negative effect on IL-31 serum levels (*p*-value < .0001), while (B) NF amount and (C) Smix ratio exhibited a significantly positive effect, *p*-values of <.0001 and <.0073, respectively.

The ability of clove oil to reduce IL-31 levels and, hence, improve the inflammatory side effects associated with NF could also be attributed to its eugenol content. Further, eugenol is known to inhibit the nuclear factor-kappa B (NF-κB) signaling pathway, which is a crucial step in preventing the transcription of cytokines and diminishing inflammation signs (Zhang et al., 2013). Moreover, compounds like eugenol that display antioxidant activity are able to modify oxidative stress and might indirectly participate in decreasing the production of inflammatory mediators. Therefore, eugenol is considered to be more effective in decreasing inflammation through its combined anti-inflammatory and antioxidant actions (Barboza et al., 2018).

The positive effect of NF and CPC on IL-31 levels might be associated with mild inflammatory side effects in patients, which might affect their adherence to such drugs to some extent. Figure 5 displays the main effect diagrams, 3D surface response and contour plots, revealing the effects of the studied factors on IL-31 serum levels.

### 3.4. Optimization of NF-loaded nanoemulsion formulations

**Figure 6.**
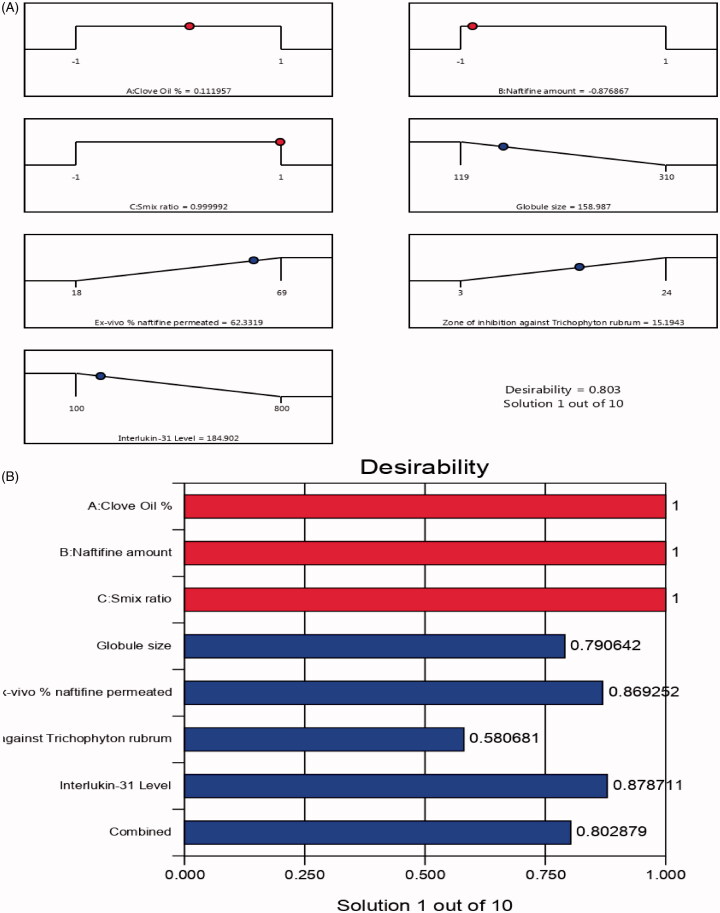
Desirability ramp and bar chart for optimization. (A) Desirability ramp shows the levels for independent variables and predicted values for the responses of the optimum formulation. (B) Bar chart shows the desirability values for the combined responses.

From the previous data, an optimum NE formulation was developed using the most suitable properties. Design Expert^®^ indicated several solutions that could be used as various combinations of the independent variables’ levels. The optimum formulation was found to be composed of 14% clove oil and 12.5 mg NF with a Smix ratio of 3:1. The formula resulted in a globule size of 161 nm, *ex vivo* % naftifine permeated of 64%, IL-31 value of 180 pg/mL, and zone of inhibition against *Trichophyton rubrum* of 16 mm with 0.8030 desirability. It was noteworthy that optimum formulation had a much smaller zone of inhibition against *Trichophyton rubrum* (16 mm) compared to that of commercial cream (26 mm). Figure 6 presents the desirability plot, and Table 5 indicates that the observed and predicted values of the optimum formulation parameters were in close agreement with no major differences (*p* > .05), proving the model’s good predictability and validity.

**Table 5.** Actual and experimental values of the optimized nanoemulsion formulation.

Solution	Clove oil %	NF amount (mg)	Smix ratio	Droplet size (nm	*Ex vivo* % naftifine permeated	Zone of inhibition against *Trichophyton rubrum* (mm)	Interleukin-31 Level (pg/mL)	desirability
Predicated value	14	12.5	3:1	158.7	62.3	15.1	185	0.80
Experimental value	14	12.5	3:1	161.0	64.0	16.0	180	0.80

### 3.5. Check point analysis

**Figure 7.**
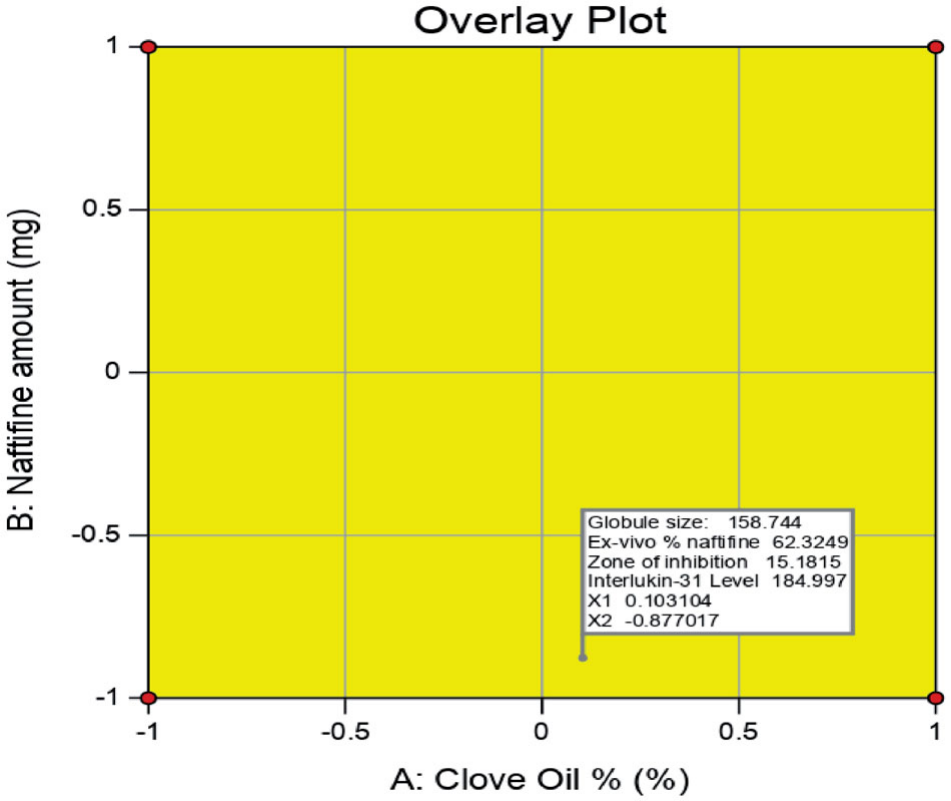
Overlay plot for the optimal NF-CO SNEDDS region determination.

Expected and adjusted *R*^2^ values of the measured responses were in close agreement, validating the significant prediction capability of the design. In addition, experimental/predicted ratios with a percentage error below 10% and acceptable residuals were observed between the experimental and predicated responses, showing the lack of curvature in the responses and validity of the model. The results are presented in Table 6, and Figure 7 illustrates the overlay plot for the optimal region.

**Table 6.** Composition, actual, and predicted responses of the optimal nanoemulsion formulation.

Factor	Optimal value	Response Variable	Actual value	Predicted value	% Prediction error^a^
A:Clove oil %	14	Droplet size (nm)	161	158.7	0.006
B:NF amount (mg)	12.5	*Ex vivo* % naftifine permeated	64	62.3	0.026
C:Smix ratio	3:1	Zone of inhibition against *Trichophyton rubrum* (mm)	16	15.1	0.056
		Interleukin-31 Level (pg/mL)	180	185	−0.027

^a^Calculated as [Actual − predicted/Actual] × 100.

### 3.6. Zeta potential measurements of optimum formulation

The optimized NF loaded nanoemulsion formulation acquired a ZP value of 28.31 ± 1.37 mV, further verifying the good stability of the developed formulation due to considerable repulsion between globules as predicted from such a large value. Furthermore, such a positive value will help in enhancing retention of NF in the skin and its effect through binding with the negatively charged phospholipid moieties of the skin. Notably, the observed large + ve ZP value might be ascribed to the use of cetylpyridinium chloride as a surfactant, which has a high positive charge.

### 3.7. *Ex vivo* permeation study of optimum formulation

The optimized nanoemulsion formulation exhibited better skin permeation parameters compared to a commercial product. As seen in Table 7, the cumulative amount of permeated NF, steady state flux, permeability, and diffusion coefficients were improved by 2-, 3-, 5.75-, and 2.74-fold, respectively, in the optimum formulation compared to the commercial cream. Such enhancement in NF permeation in the case of nanoemulsion formulation could be due the NE components. As previously discussed, the main component of clove oil, eugenol, plays a pivotal role in improving skin penetration due to its ability to cause some disruption in the SC layer and deactivate its barrier properties.

**Table 7.** Permeation parameters of optimum NF-CO-SENDDs.

Permeation parameters	Optimized formulation	Commercially available cream
Cumulative amount permeated (μg/cm^2^)	3300 ± 423	1633 ± 212
Steady state flux, Jss, (μg/cm^2^ min)	9.23 ± 1.33	3.12 ± 0.62
Permeability coefficient, P, (cm/min)	1.30 × 10^−3^	0.22 × 10^−3^
Diffusion coefficient, D, (cm^2^/min)	16.12 × 10^−5^	5.87 × 10^−5^
Enhancement factor (EF)	2.02	

### 3.8. Skin sensitivity test

As seen in Table 8, the developed optimum NF-CO-SENDDs formulation was tolerated much better by rat skin than the commercial product. The reason behind such compatibility of NE formulation with the skin might be attributed to its clove oil content, which provides a good anti-inflammatory effect, as previously reported. These results are in good accordance with previously discussed results concerning IL-31 levels.

**Table 8.** Scoring of skin sensitivity test results of optimum NF-CO-SENDDs.

	Itching	Erythema	Papule	Flakiness	Dryness	Average Sensitivity Score
Control	0	1	0	0	0	1/20
Optimized formulation	1	0	0	0	1	2/20
Commercial cream	3	3	2	2	2	12/20

0: no sign of inflammation; 1: mild; 2: moderate; 3: severe.

### 3.9. Stability study

After storing the optimized formulation for 48 h subjected to three freeze-thaw cycles between −20 °C and +25 °C, no major differences were observed from the freshly prepared formula, as observed in Table 9. Such results confirm the adequate stability of the developed optimum NF-CO-SENDD, which can thus be properly used and stored.

**Table 9.** Physical stability parameters of stored and freshly prepared NF-CO-SENDDs.

	Emulsification ability	Precipitation	pH	Globule size (nm)	PDI
Freshly prepared optimized formulation	Simultaneous emulsification	No	6.64 ± 0.21	161 ± 6	0.21
Optimized formulation after stability test	Simultaneous emulsification	No	6.49 ± 0.19	164 ± 4	0.23

## 4. Conclusions

In the current investigation, a Box–Behnken design was adopted to develop and optimize a clove oil-based nanoemulsions loaded with NF for the topical treatment of tinea. The optimal clove oil % that was used in NE formulation ranged between 10 and 17%, and the produced NF-CO-SENNDs gained an adequate droplet size between 119 and 310 nm, indicating optimal NE formation. The statistical design confirmed the synergistic effect of clove oil and NF in the treatment of fungal infections and proved the clove oil’s anti-inflammatory effect that can reverse the side effects of NF. The optimized formulation composed of 14% clove oil, 12.5 mg Naftifine, and prepared with an Smix ratio equaling 3:1, exhibited good antifungal and anti-inflammatory activity, achieving up to 2-, 3-, 5.75-, and 2.74-fold increases in the amount of permeated NF, steady state flux, permeability, and diffusion coefficients, respectively, compared with a commercial product. Furthermore, the optimized formulation gained an adequate zeta potential value of 28.31 ± 1.37 mV and showed reasonable thermodynamic stability together with no or mild signs of skin sensitivity. Collectively, the designed nanoemulsion containing clove oil and naftifine presents a promising topical delivery systems for the management of tinea.
